# Electrochemical and structural characterization of *Azotobacter vinelandii* flavodoxin II

**DOI:** 10.1002/pro.3236

**Published:** 2017-08-30

**Authors:** Helen M. Segal, Thomas Spatzal, Michael G. Hill, Andrew K. Udit, Douglas C. Rees

**Affiliations:** ^1^ Division of Chemistry and Chemical Engineering Howard Hughes Medical Institute, California Institute of Technology Pasadena California 91125; ^2^ Division of Chemistry Occidental College Los Angeles California 90041

**Keywords:** flavodoxin, nitrogenase, X‐ray crystallography, electrochemistry

## Abstract

*Azotobacter vinelandii* flavodoxin II serves as a physiological reductant of nitrogenase, the enzyme system mediating biological nitrogen fixation. Wildtype *A. vinelandii* flavodoxin II was electrochemically and crystallographically characterized to better understand the molecular basis for this functional role. The redox properties were monitored on surfactant‐modified basal plane graphite electrodes, with two distinct redox couples measured by cyclic voltammetry corresponding to reduction potentials of −483 ± 1 mV and −187 ± 9 mV (vs. NHE) in 50 mM potassium phosphate, 150 mM NaCl, pH 7.5. These redox potentials were assigned as the semiquinone/hydroquinone couple and the quinone/semiquinone couple, respectively. This study constitutes one of the first applications of surfactant‐modified basal plane graphite electrodes to characterize the redox properties of a flavodoxin, thus providing a novel electrochemical method to study this class of protein. The X‐ray crystal structure of the flavodoxin purified from *A. vinelandii* was solved at 1.17 Å resolution. With this structure, the native nitrogenase electron transfer proteins have all been structurally characterized. Docking studies indicate that a common binding site surrounding the Fe‐protein [4Fe:4S] cluster mediates complex formation with the redox partners Mo‐Fe protein, ferredoxin I, and flavodoxin II. This model supports a mechanistic hypothesis that electron transfer reactions between the Fe‐protein and its redox partners are mutually exclusive.

## Abstract 2

PDB Code(s): 5K9B

## Introduction

The formation of bioavailable ammonia from the reduction of atmospheric dinitrogen is an important reaction in the nitrogen cycle. Nitrogenase is the enzyme that catalyzes this process using energy derived from ATP hydrolysis to form two molecules of NH_3_ and one molecule of H_2_ from N_2_.^1^, ^2^, ^3^ This enzyme consists of two proteins: the Fe‐protein and the MoFe‐protein. The MoFe‐protein provides the active site for dinitrogen reduction while the Fe‐protein mediates the ATP dependent transfer of electrons to the MoFe‐protein. Although it is clear that reduction of dinitrogen to ammonia requires the delivery of multiple electrons to the active site of the MoFe‐protein from the reduced iron‐sulfur cluster of the Fe‐protein, the biological source of these electrons remains less well defined. Dithionite typically serves as the electron donor for *in vitro* studies, whereas flavodoxins and ferredoxins have been implicated as the physiological reductants of the Fe‐protein.^4^, ^5^, ^6^, ^7^, ^8^, ^9^, ^10^ In *Azotobacter vinelandii*, flavodoxin II and ferredoxin I, the products of the *nifF* and *fdxA* genes, respectively, have been shown to serve as electron donors to nitrogenase, although other proteins can also serve this function.^9^

There is redundancy in the biological electron donors of the nitrogenase Fe‐protein in *A. vinelandii*; however, four observations suggest that flavodoxin II is one of the predominant reductants. First, deletion of *Klebsiella pneumoniae nifF* resulted in the inability to fix nitrogen, suggesting that at least in some bacteria, flavodoxin II performs a critical role in donating electrons to nitrogenase.^11^ Second, the expression level of flavodoxin II in the cell is upregulated under nitrogen fixing conditions.^12^ Third, the Fe‐protein and nitrogenase‐associated flavodoxin can form a complex,^6^, ^8^, ^13^ and electron transfer from the reduced flavodoxin to the Fe‐protein is rapid with rates exceeding 10^6^ M^−1^ s^−1^. Finally, *in vitro* nitrogenase activity assays using flavodoxin II as a source of electrons have demonstrated that this flavodoxin can serve as an electron donor to nitrogenase.^8^, ^14^ Furthermore, evidence has been presented that with flavodoxin as the biological reductant, the specific activity of the MoFe‐protein increases,^15^ and the ATP/2e^–^ ratio decreases to 2 from a value of about 4 typically observed with dithionite.^14^, ^16^ These observations suggest that energy utilization is more efficient with the physiological reductant than with dithionite.

*A. vinelandii* flavodoxin II is a prototypical long chain flavodoxin with a non‐covalently bound flavin mononucleotide (FMN) cofactor.^17^ This cofactor can exist in three different oxidation states: oxidized quinone, one‐electron reduced semiquinone, and two‐electron reduced hydroquinone. For free FMN in solution,^18^ the relevant reduction potentials of the quinone/semiquinone and semiquinone/hydroquinone states are *E*_2_ ∼ −325 mV and *E*_1_ ∼ −150 mV versus NHE, respectively, at pH 7. Consequently, free FMN cycles between the oxidized quinone and two‐electron reduced hydroquinone due to rapid disproportionation of the semiquinone state in aqueous solutions. When bound by flavodoxin, however, the semiquinone form of FMN is stabilized, which allows for the sequential transfer of one electron at low potentials. This behavior is reflected in a positive shift in the quinone/semiquinone potential (*E*_2_) to about −250 to −100 mV, and a negative shift in the semiquinone/hydroquinone potential (*E*_1_) to about −500 to −400 mV.^19^ The flavodoxin bound FMN cycles between the semiquinone and the hydroquinone states in most low‐potential redox reactions.^17^ Studies of the pH dependence of the flavodoxin potentials suggest that reduction of the oxidized protein to form the semiquinone is accompanied by the uptake of 1 H^+^, while reduction of the semiquinone to form the hydroquinone state has a more complicated pH dependence with a proton‐coupled electron transfer process observed below pH 6.5.^20^, ^21^

In this article, a series of X‐ray crystallography and electrochemistry experiments were performed to better characterize the structural and thermodynamic properties of native *A. vinelandii* flavodoxin II relevant to the function of this protein in nitrogenase mediated biological nitrogen fixation. For this purpose, a method was adapted to determine the redox properties of *A. vinelandii* flavodoxin II on surfactant‐modified basal plane graphite electrodes. This is a novel method to study the redox properties of flavodoxins in an environment that potentially reflects features of the flavodoxin II‐Fe‐protein complex that may not be captured in bulk solution. In addition, a 1.17 Å resolution structure of the flavodoxin purified from *A. vinelandii* established the underlying structural similarity to the recombinant protein reported previously.^19^ Although reported previously,^22^, ^23^, ^24^, ^25^, ^26^ no phosphodiester linkage between amino acid side chains was observed in this flavodoxin II isolated from *A. vinelandii*. This was the last unsolved structure of wildtype nitrogenase electron transfer proteins isolated from *A. vinelandii*, and allowed for the modeling of the interactions between the Fe‐protein and these proteins. Docking studies suggest a common interaction site between the Fe‐protein and flavodoxin II, ferredoxin I, and the MoFe‐protein.

## Results and Discussion

### Electrochemistry of flavodoxin II on didodecyldimethylammonium bromide‐modified basal plane graphite electrodes

The redox properties of *Azotobacter vinelandii* flavodoxin II have previously been studied using both a direct electrochemical method and by EPR‐monitored redox titrations^12^, ^21^, ^22^, ^27^, ^28^, ^29^ (Supporting Information Table S1). Since, as noted above, the reported *E*_1_ and *E*_2_ potentials of *Azotobacter vinelandii* flavodoxin II exhibit considerable variability, we developed a new method for monitoring the redox properties of this protein on surfactant‐modified basal plane graphite electrodes.

Surfactant‐modified electrodes have been extensively used to study the redox chemistry of heme proteins, including myoglobin and cytochrome P450.^30^, ^31^, ^32^ In this technique, synthetic or natural surfactants form ordered films of stacked bilayers on an electrode, akin to biological membranes found in nature. Although these films were originally developed to study the redox behavior of membrane proteins, the observation of direct electron transfer between myoglobin and an electrode modified with these films suggested that they could be used as a general tool for monitoring the redox chemistry of water soluble proteins. Since that time, a number of studies have been performed that examine the redox properties, the spectroscopic characteristics, and the electrocatalytic activity of proteins in these films.^33^, ^34^

In this study, the redox behavior of *Azotobacter vinelandii* flavodoxin II was characterized on basal plane graphite electrodes (BPGE) coated with a film of the surfactant didodecyldimethylammonium bromide (DDAB). This film was chosen because the positive charge on the head‐group of the DDAB may help mimic potential electrostatic interactions in electron transfer complexes between flavodoxin and the nitrogenase Fe‐protein. In addition, electron transfer between the flavodoxin and the surfactant coated graphite electrode is anticipated to decrease the solvent accessibility of FMN, which is hypothesized to occur during formation of a flavodoxin II ‐ Fe‐protein complex. As a technical point, this study constitutes one of the first examples of the electrochemical characterization of a protein that contains a flavin cofactor with this technique,^31^ thus expanding the applicability of this approach to studies of the redox behavior of flavoproteins.

**Figure 1:**
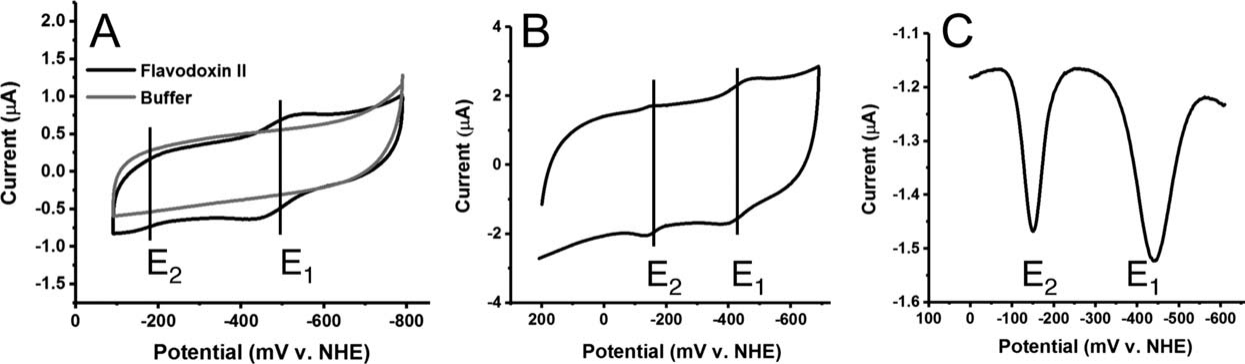
Flavodoxin II on Basal Plane Graphite Electrodes Modified with DDAB. (A) Cyclic voltammograms in the presence (black) or absence (gray) of 200 µM native *A. vinelandii* flavodoxin II in 50 mM potassium phosphate, pH 7.5, 150 mM NaCl. *E*_1_ and *E*_2_ can be observed on this voltammogram at −483 ± 1 and −187 ± 9 mV, respectively. Standard errors were calculated from 3 separate cyclic voltammograms acquired with 3 different electrodes and 2 different batches of proteins. The voltammogram was acquired at a scan rate of 10 mV/s. (B) The DDAB‐modified basal plane graphite electrode was soaked for twenty minutes in a 200 μM solution of recombinant flavodoxin II purified from *E. coli* in 50 mM potassium phosphate, pH 7.5, 150 mM NaCl. The electrode was transferred to 10 mM phosphate buffer, pH 7, and a cyclic voltammogram was acquired at a scan rate of 50 mV/s. *E*_1_ and *E*_2_ values are −427 ± 3 mV and −151 ± 3 mV, respectively. (C) Square wave voltammogram of flavodoxin II on DDAB‐modified basal plane graphite electrodes showing the reduction potentials of *E*_1_ and *E*_2_.

There were two redox active species present when cyclic voltammograms were acquired with the working electrode in a 200 μM flavodoxin II solution at pH 7.5 in 50 mM potassium phosphate buffer with 150 mM NaCl: a lower potential redox species with a midpoint potential of −483 ± 1 mV and a higher potential redox species with a midpoint potential of −187 ± 9 mV [Fig. 1(A)]. The lower potential species underwent a reversible redox process whereas the higher potential redox species appeared to be quasi‐reversible with oxidation occurring more readily than reduction. These redox processes were observed immediately on submersion of the DDAB‐modified electrode in flavodoxin II solution. There was no signal associated with either redox process on unmodified basal plane graphite electrodes or on DDAB‐modified electrodes placed in buffer [Fig. 1(A), Supporting Information Figure S1]. Recombinant flavodoxin II purified from *E. coli* rather than from the native organism had the same redox‐active species observed by cyclic voltammetry as the native protein and similar midpoint potentials, which suggested that both native and recombinant flavodoxin II have comparable redox activity [Fig. 1(B,C)].

The lower potential species was assigned to the hydroquinone/semiquinone redox couple based on the similarity to previously measured midpoint potentials. Identification of the higher potential redox‐active species was more challenging since both the magnitude and reversibility of this peak were variable. This was especially evident during the pH titrations which showed a more pronounced signal for the higher potential redox active species at low pH where the protein is less stable. This observation initially supported assignment of the higher potential peak as the two‐electron hydroquinone/quinone couple of FMN in solution (Supporting Information Fig. S2), which is similar in potential to the quinone/semiquinone couple of flavodoxin II.^20^, ^21^, ^35^ In view of the results of the pH titrations discussed below, it is likely, however, that a majority of the observed higher potential species is the semiquinone/quinone couple of the flavodoxin under conditions that favor flavodoxin stability. As discussed by Heering and Hagen,^36^ slow electrode kinetics of the one‐electron reduced flavodoxin II semiquinone could explain why the higher and lower potential redox peaks are not present at a 1:1 ratio on the cyclic voltammogram. An analysis of the kinetics indicates that the interaction with the DDAB surfactant does interfere with the free diffusion of flavodoxin II at the electrode (Supporting Information Fig. S3).

In addition to analyzing the redox activity of flavodoxin II directly in a solution of this protein, the DDAB‐modified basal plane graphite electrodes could be loaded with the protein, and the electrode moved to a phosphate buffer solution. The protein remained stable in the surfactant film during the course of the experiment on the order of hours. Under these conditions (10 mM potassium phosphate buffer, pH 7), the midpoint potentials of the two redox active species were shifted to −454 ± 7 mV for the lower potential redox species and −164 ± 12 mV for the higher potential redox species (Supporting Information Fig. S3). This method was used for all subsequent scan rate and pH experiments.

**Figure 2:**
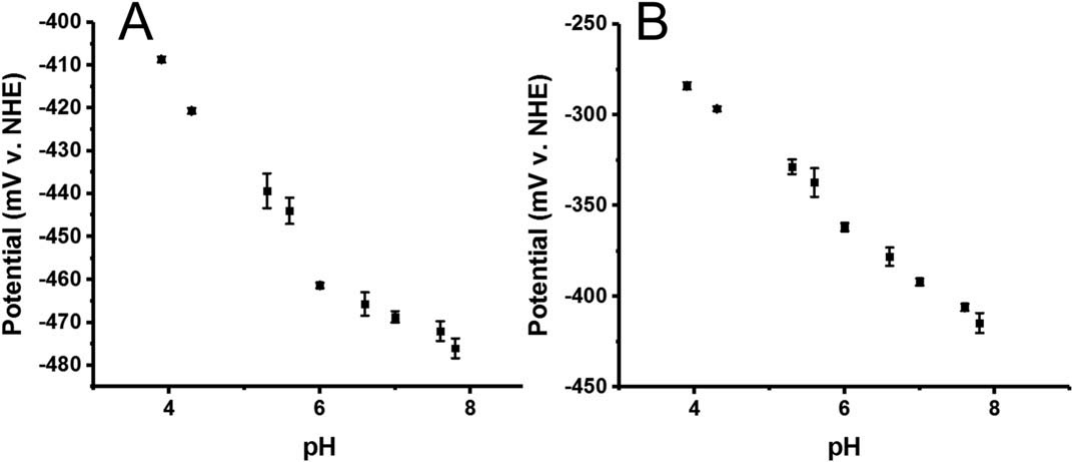
pH Titration of Flavodoxin II. (A) Dependence of the midpoint potential of the lower potential redox species (semiquinone/hydroquinone potential *E*_1_) on the solution pH. Each data point is the average of three separate experiments, with the standard error of the mean indicated. The slope of the linear portion of this curve at low pH is 23 mV/pH unit. (B) Dependence of the midpoint potential of the higher potential redox species (quinone/semiquinone couple *E*_2_) on the solution pH. Each data point is the average of three separate experiments, with the standard error of the mean indicated. The slope of the linear portion of this curve was 34 mV/pH unit.

The pH dependence of the midpoint potential of flavodoxin II was also assessed on DDAB‐modified basal plane graphite electrodes to further characterize this system. The pH range evaluated in these studies was from pH 3.9–7.8. Consistent with previous studies using carbon electrodes modified with neomycin, there was a strong increase in potential with decreasing pH from pH 4–pH 6. When fit with the Nernst equation, this data was consistent with a proton coupled electron transfer reaction, with the pK_a_ of this proton estimated to be 6.1 [Fig. 2(A)]. Analysis of the higher potential peak suggested that the pH dependence of the potential of this species was approximately linear [Fig. 2(B)]. FMN has a pK_a_ of about 6.4–6.7 for the protonation of reduced molecules.^21^ Thus, the observed pH titration of the higher potential redox process is not consistent with the behavior of free FMN. A more likely explanation is that the observed redox process corresponds to the quinone/semiquinone couple where in addition to the uptake of a proton to give the neutral semiquinone, there is also a redox linked protonation associated with the semiquinone form.^37^ The pH titrations indicate that the potentials of both the hydroquinone/semiquinone and the semiquinone/quinone couples can be measured with this electrochemical method.

### X‐ray crystallography

**Figure 3:**
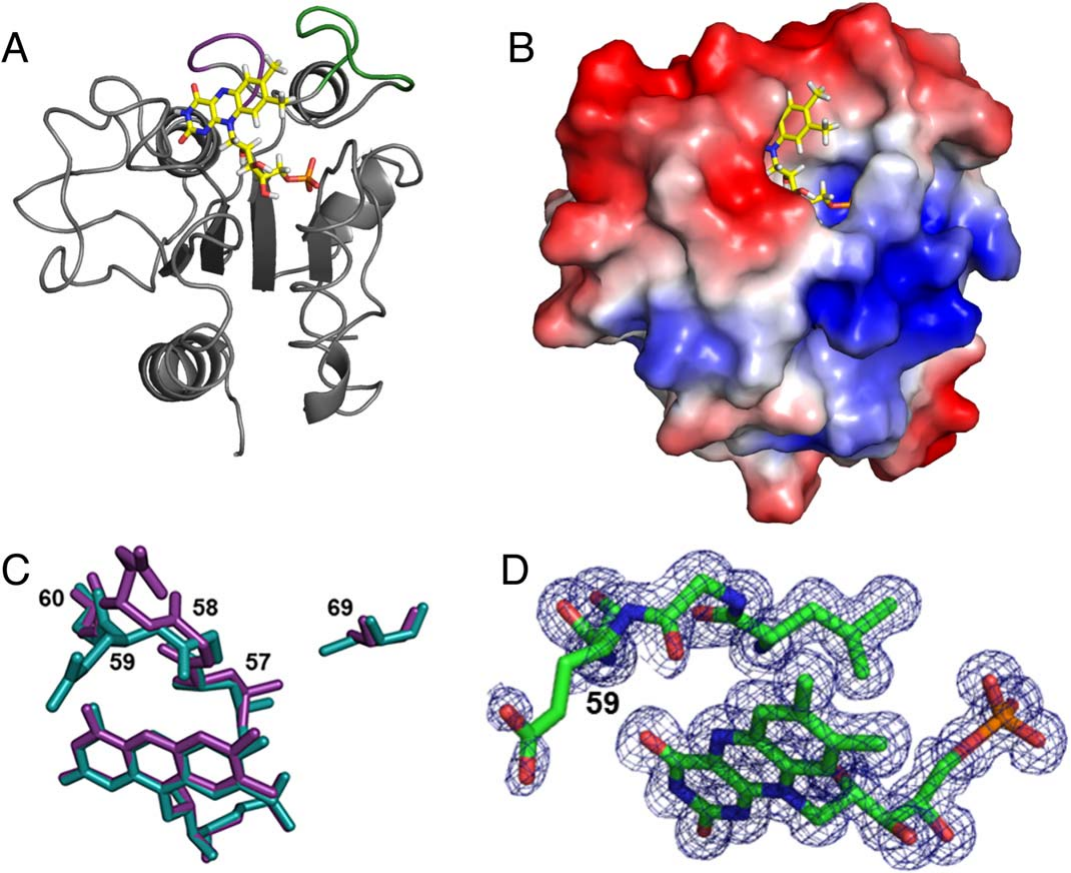
Structure of Flavodoxin II. (A) Ribbon structure of wildtype *A. vinelandii* flavodoxin II. The FMN cofactor is depicted as a ball‐and‐stick model with yellow bonds. The eight amino acid stretch proposed to be involved in mediating the flavodoxin Fe‐protein interaction is shown in green. The 50 s loop involved in interactions with FMN that are important for modulating the redox behavior of this cofactor is highlighted in purple. (B) Electrostatic surface potential diagram of flavodoxin II showing the two charged surfaces proposed to interact with the Fe‐protein. The identity of the negatively charged amino acid residues on flavodoxin II at the interaction site (red surface) are Asp68, Glu70, Glu72, Glu75, Glu76, Asp98, Glu104, Glu108, Glu12, Asp131, Glu134, and Glu136. The identity of the positively charged amino acid residues on flavodoxin II at the interaction site (blue surface) are Lys13, Arg15, Lys16, Lys19, and Arg38. (C) Overlay of the structure of oxidized wildtype flavodoxin II (cyan) and the structure of the Cys69Ala mutant of flavodoxin II (purple) (PDB ID: 1YOB). The amino acid side chains of residues 57–60 are shown along with the FMN cofactor to illustrate the variation observed in this region between the wildtype and mutant structure. (D) Close‐up of the electron density around the FMN cofactor and Gly58‐Glu59 to illustrate the weak electron density around these residues.

The crystal structure of *A. vinelandii* flavodoxin II was solved at 1.17 Å resolution by molecular replacement using the model of the Cys69Ala mutant of *A. vinelandii* flavodoxin II (PDB ID 1YOB^19^). Following refinement (with statistics presented in Supporting Information Table S2), the root mean square displacements between the wildtype and Cys69Ala variant are 0.45 Å and 0.97 Å based on superpositions of the Cα and all atoms, respectively. The overall three‐dimensional structure of flavodoxin II [Fig. 3(A)] reflects the prototypical α/β‐architecture of long chain flavodoxins,^17^ including the insertion of 22‐amino acids in the fifth β‐strand, which is characteristic of long chain flavodoxins.^38^ No phosphodiester modification was observed in the structure, as has been reported previously to be present in *A. vinelandii* flavodoxin II;^22^, ^23^, ^24^, ^25^, ^26^ perhaps reflecting strain variations or differences in growth conditions. The *Azotobacter* flavodoxin II also has an insertion of eight amino acid residues (amino acids 64–71) near the FMN cofactor that is generally found in nitrogenase‐associated flavodoxins; these residues have been proposed to be involved in complex formation with the Fe‐protein of nitrogenase.^19^ The electrostatic surface of flavodoxin II surrounding this region [Fig. 3(B)] reveals striking features of charge asymmetry that may be relevant for interaction with electron transfer partners.

The major difference between the crystal structure of the Cys69Ala flavodoxin II mutant^19^ and wildtype flavodoxin II is the identity of the side chain position at residue 69 [Fig. 3(C)]. Residue 69 is within the surface exposed loop (residues 64–71) of flavodoxin II proposed to interact with the Fe‐protein, and thus the identity of this residue may influence formation of the protein complex. In wildtype flavodoxin II, the Cys69 side chain points toward the interior of the protein near the FMN cofactor, so that formation of a disulfide‐bridged flavodoxin II dimer would require repositioning of this loop.^21^ The SG of Cys69 is in van der Waals contact (3.51 Å) with the CD1 methyl group of Leu57, and the presence of this non‐covalent interaction is likely reflected in the variations in the positioning of the FMN and residues 57–60 observed in this region between the wildtype and mutant structures (Fig. 3).

The conformations of residues 56–60 of flavodoxin II have been shown to be sensitive to the oxidation state of FMN and help set the midpoint potentials of the redox couples of this cofactor. The peptide bond between residues 58 and 59 is particularly noteworthy as it has been observed to undergo *cis‐trans* isomerization and flipping of the peptide bond orientation in response to mutagenesis and FMN reduction.^39^ In general, the residue at position 58 (glycine in flavodoxin II) is in the “O‐down” conformation in the oxidized quinone form with the carbonyl of the amide backbone pointing away from FMN.^40^ On reduction to the semiquinone state, the amide bond flips such that the carbonyl is placed in an “O‐up” conformation that is able to hydrogen bond with the protonated N5 position of FMN. The ambiguous electron density for the peptide bond between Gly58‐Glu59, as well as weak density for the side chain of Glu59 in the wildtype flavodoxin II structure [Fig. 3(D)], is indicative of a mixture of conformations that could not be cleanly resolved into components such as *cis* “O‐down,” *trans* “O‐down” and *trans* “O‐up” conformations as reported previously for a *Clostridial* flavodoxin.^39^ Given the yellow color of the crystals, it is very likely that they exhibit an oxidized state, but the presence of some residual reduced forms from the anaerobic purification and/or photoreduction in the X‐ray beam cannot be dismissed due to weak electron density for Gly58‐Glu59.

### Model of the flavodoxin—Fe‐protein complex

**Figure 4:**
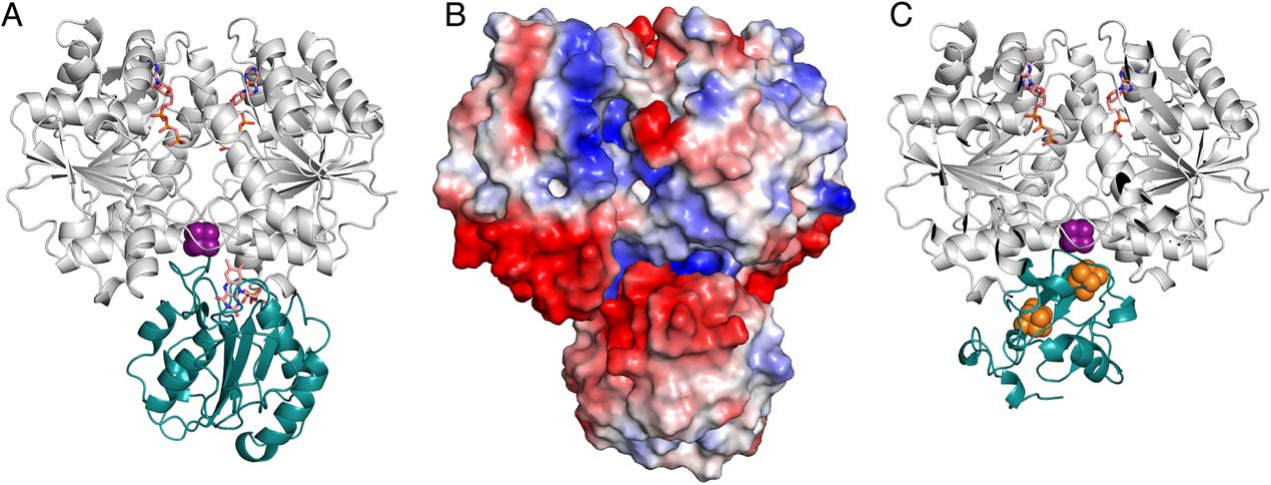
Model of the Flavodoxin II‐Iron Protein Complex. (A) Ribbon diagram of the model of flavodoxin II (teal) binding to the Fe‐protein (gray) bound to MgAMP‐PCP. The FMN cofactor and the AMP‐PCP molecules are shown in orange. The iron‐sulfur cluster is shown in purple. (B) Electrostatic surface potential diagram of the MgAMP‐PCP bound Fe‐protein flavodoxin II complex. (C) Ribbon diagram of the model of ferredoxin I (teal) binding to the Fe‐protein (gray) bound to MgAMP‐PCP. The iron‐sulfur clusters bound by ferredoxin I are shown in orange. The iron‐sulfur cluster bound by the Fe‐protein is shown in purple. Ferredoxin I and flavodoxin II are predicted to bind to the same region of the Fe‐protein.

The ClusPro2.0 algorithm^41^, ^42^, ^43^, ^44^ was used to construct a model of the electron transfer complex formed between wildtype flavodoxin II and the Fe‐protein. The MgATP‐bound form of the Fe‐protein is the physiologically relevant state for both reduction by flavodoxin II, as well as for electron transfer to the MoFe‐protein. Unfortunately, this form of the isolated Fe‐protein has not been structurally characterized. Consequently, the ClusPro algorithm was used to generate possible models of the interaction of flavodoxin II with the nucleotide‐bound forms of the Fe‐protein that had originally been solved in complex with the MoFe‐protein. The preferred model generated by this algorithm [Fig. 4(A)] suggests that the Fe‐protein and flavodoxin II can associate such that the FMN cofactor and the Fe‐S cluster are positioned within ∼5 Å. This docking model is similar to that previously described for the Cys69Ala flavodoxin II mutant and nucleotide‐free Fe‐protein^45^ also generated using the ClusPro2.0 algorithm. Notably, these models predict that flavodoxin II binds to the Fe‐protein at the same site as the MoFe‐protein during nitrogenase complex formation.

This model emphasizes the role of electrostatic interactions with two strongly charged regions on the flavodoxin facilitating this binding mode, including the eight residue insertion found in nitrogenase‐associated flavodoxins (Fig. 4). Specifically, in this model of complex formation, negatively charged residues of the flavodoxin surrounding FMN interact with positively charged amino acids surrounding the iron‐sulfur cluster of the Fe‐protein. There is also a region of the flavodoxin that has a net positive charge, which can interact with a negatively charged region of the Fe‐protein during complex formation [Fig. 4(A)]. While electrostatic interactions are anticipated to play critical roles in the electron transfer reaction between Fe‐protein and flavodoxin based on their interactions with other redox partners,^46^, ^47^, ^48^ it is worth noting, however, that there is no experimental evidence for this behavior in the *A. vinelandii* Fe‐protein ‐ flavodoxin system. Indeed, the reaction between the Fe‐protein and NifF Fld of *Rhodobacter capsulatus* has been reported to be insensitive to ionic strength,^6^ which can be used to assess the contributions of electrostatic interactions.

To determine whether there is a conserved region where the Fe‐protein interacts with electron transfer partners, models were constructed with ClusPro for the docking of Fe‐protein (PDB ID: 4WZB) to both the MoFe‐protein (PDB ID: 3U7Q) and ferredoxin I [PDB ID: 6FDR; Fig. 4(B)]. The former calculation serves as a validation of the docking procedure as it identifies the crystallographically observed binding interaction between the *A. vinelandii* MoFe‐protein and Fe‐protein.^49^ These models support the presence of a common binding site surrounding the Fe‐protein [4Fe:4S] cluster that is used to interact with the physiological electron transfer partners (Supporting Information Fig. S4). This behavior is consistent with the mechanistic hypothesis that electron transfer reactions between the Fe‐protein and its redox partners are mutually exclusive, as deduced from the kinetics studies of nitrogenase that are consistent with an obligatory dissociation of the MoFe‐protein and Fe‐protein after each cycle of electron transfer.^50^, ^51^

## Conclusion

The *in vivo* roles and physiological consequences of different electron donors to nitrogenase have not been extensively studied.^9^ A comprehensive understanding of these physiological reductants will be important for understanding how nitrogenases work in cells. Evidence for the reduction of the *A. vinelandii* nitrogenase Fe‐protein to the all‐ferrous oxidation state by flavodoxin II suggests that the electron transfer reaction between these two proteins could be important for *in vivo* nitrogenase activity.^14^, ^16^, ^52^ Furthermore, reports that the specific activity of the Fe‐protein is increased 50% with flavodoxin as an electron donor relative to the *in vitro* assay with dithionite as the reductant,^15^ and that the all ferrous form of the Fe‐protein supports substrate reduction with half the ATP consumption as with dithionite,^14^, ^16^ suggest potentially profound metabolic consequences of the electron donor for biological nitrogen fixation. The electrochemical and structural characterization of the physiological electron donors provides the foundation for more detailed analyses of the mechanism of Fe‐protein reduction by the physiological reductant flavodoxin II. As a starting point, the electrochemical characterization of flavodoxin II on basal plane graphite electrodes developed in this work provides a new approach for such studies in an environment that may be more representative of the physiological electron transfer reaction than solution‐based methods.

## Materials and Methods

### Azotobacter vinelandii flavodoxin II purification

Cell growths and purifications of *Azotobacter vinelandii* nitrogenase proteins (including flavodoxin II) were performed as described previously.^53^, ^54^ Recombinant flavodoxin II expressed in *E. coli* was purified following published reports.^19^, ^55^ Detailed protocols are provided in the Supporting Information.

### Preparation of didodecyldimethylammonium bromide films on basal plane graphite electrodes

Cylindrical (0.2 cm^2^) basal‐plane graphite electrodes were used for electrochemistry. Prior to experiments, electrodes were polished sequentially with 0.3 micron and 0.05 micron alumina resin. The electrodes were cleaned by sonication for 10 min, then were dried with a heat gun. 5 μL of 10 mM DDAB in deionized water was deposited onto the electrode surface. The DDAB solution was sonicated for 45 minutes prior to being deposited to the electrode. The films dried slowly overnight on to the electrode under a beaker to provide a closed environment.

### Electrochemistry with DDAB‐modified BPGE

All experiments were performed in a glovebox that had a nitrogen atmosphere. Dioxygen was removed from buffers used in electrochemistry experiments by iterative cycles of vacuum followed by filling with argon. Protein was incorporated into the film in two ways. First, the protein solution was put in the central compartment of a two‐compartment electrochemical cell. The DDAB‐modified basal plane graphite electrode was placed in the protein solution along with the platinum auxiliary electrode. The solid state Ag/AgCl reference electrode (Warner Instruments) was connected to the cell with the working electrode via a Luggin capillary. Alternatively, the electrode was soaked in a 200 μM flavodoxin II solution for twenty minutes. Then, the electrode was transferred to phosphate buffer (10 m*M* potassium phosphate, pH 7) along with a platinum auxiliary electrode. All measurements were made with a CH Instruments potentiostat, and the data were analyzed with CH Instruments software.

### pH titration

The DDAB‐modified basal plane graphite electrode was soaked in a 200 μ*M* flavodoxin II solution for twenty minutes. Experiments were run in the following buffers: 10 m*M* potassium phosphate (pH 7.9), 10 m*M* potassium phosphate (pH 7.6), 10 m*M* potassium phosphate (pH 7), 10 m*M* potassium phosphate (pH 6.6), 10 m*M* potassium phosphate (pH 6), 10 m*M* sodium phosphate (pH 5.6), 10 m*M* sodium phosphate (pH 5.4), 10 m*M* sodium acetate (pH 4.3), and 10 m*M* sodium acetate (pH 3.9). The electrodes were incubated for ten minutes in the buffer solution prior to square wave voltammogram acquisition.

### Crystallization and data collection

Flavodoxin II was crystallized using the sitting drop vapor diffusion method in an anaerobic chamber containing a 90% Ar/10% H_2_ atmosphere. The reservoir solution contained 23–28% PEG 3350 (v/v), 0.15–0.3 M MgCl_2_·6H_2_O, and 100 mM Tris‐HCl, pH 8.5. Crystals were obtained between 3 and 6 months after setting up the crystallization experiment. The flavodoxin crystals were cryo‐protected by transferring the crystals into a 5 μL drop of 7% 2‐methyl‐2,4‐pentanediol in the reservoir solution (v/v). Diffraction data were collected at an energy of 12,400 eV at the Stanford Synchrotron Radiation Lightsource (SSRL) beamline 12–2 with a Dectris Pilatus 6M detector.

### Structure solution and refinement

The data were indexed, integrated, and scaled using XDS and Scala.^56^, ^57^ Structural refinement and rebuilding was done in REFMAC5 and Coot.^57^, ^58^, ^59^ All protein structures were rendered in PyMol.^60^

## Supporting information

Supporting Information
