# Supplementary material for: Electrochemical and structural characterization of Azotobacter vinelandii flavodoxin II
Source: Protein Sci. 2017 Aug 30;26(10):1984–93. doi: 10.1002/pro.3236 (PMC5606536; doi:10.1002/pro.3236)
Supplement: Supplementary file 1 — Supporting Information [file PRO-26-1984-s001.pdf]

Supplementary Material for

Electrochemical and Structural Characterization of *Azotobacter vinelandii* Flavodoxin II

Helen M. Segal[1], Thomas Spatzal[1], Michael G. Hill[2], Andrew K. Udit[2], and Douglas C. Rees[1,*]

This PDF includes:

Supplementary Materials and Methods

Supplementary Text

Figures S1-S5

Tables S1-S2

Supplementary Materials and Methods

Azotobacter vinelandii Cell Growth and Flavodoxin II Purification. Cell growths and protein purifications were carried out as previously described for isolation of *Azotobacter vinelandii* nitrogenase [53,54]. The nitrogenase component proteins were eluted from a HiTrap Q anion exchange column (GE Healthcare) with a gradient from 150 mM NaCl to 1 M NaCl over thirty column volumes. The flavodoxin eluted from the column at conductivity values between 39 to 40 mS/cm. The protein was further purified on a size exclusion column (Superdex200, 26/60, GE Healthcare). The protein concentration was quantified by Bradford assay using a BSA standard curve.

Strain Construction for Overexpression of Flavodoxin II in E. coli. The flavodoxin II overexpression plasmid was synthesized by Genescript USA, Inc. In this plasmid, the *Azotobacter vinelandii nifF* gene was cloned into the pET21b(+) vector between the NdeI-BamHI restriction enzyme cut sites. A stop codon was included at the C-terminus of the gene so that the protein was expressed without affinity tags. The *nifF* gene was optimized for *E. coli* codon usage. The plasmid was transformed into *E. coli* BL21 (DE3) cells for overexpression of flavodoxin II.

E. coli Cell Growth and Purification of Recombinant Flavodoxin II. The protocol for purification of *Azotobacter vinelandii* flavodoxin II was adapted from previously published protocols [19,55]. The following buffers were prepared, adjusted to the correct pH at room temperature, and filtered with a glass fiber filter: anion exchange buffer A (50 mM Tris-HCl, pH 7.5, 150 mM NaCl, 2 mM dithiotreitol (DTT)), anion exchange buffer B (50 mM Tris-HCl, pH 7.5, 1 M NaCl, 2 mM DTT), and size exclusion buffer (50 mM Tris-HCl, pH 7.5, 200 mM NaCl). DTT was added to all buffers

the day of purification. Dioxygen was removed from the size exclusion column buffer prior to use in the purification by iterative cycles of vacuum followed by argon filling.

An overnight starter culture of the flavodoxin II overexpression strain supplemented with 100 µg/mL ampicillin was incubated at room temperature with shaking overnight. The starter culture was diluted 1:100 into 1 L of LB medium containing 50 µg/mL ampicillin. The cultures were grown to late exponential phase (OD_{600} ~ 0.6-0.8) at 37 °C with shaking. Overexpression of flavodoxin II was induced by addition of IPTG to a final concentration of 0.5 mM in each culture. The cultures were incubated for 16 hours at 37 °C with shaking. The cells were pelleted via centrifugation at 6238 x g for ten minutes. The cell pellets were stored at -80 °C until purification.

All lysis steps were carried out on ice at room temperature. The BL21 (DE3) cell pellets were thawed on ice. The cell pellets were re-suspended in anion exchange buffer A with a homogenizer. The lysis buffer also contained complete protease inhibitor tablets (Roche) at a concentration of one tablet per 50 mL of buffer. The cells were lysed with an Avestin Emulsiflex C5 homogenizer. The cell debris was pelleted by centrifugation in a floor centrifuge at 13,000 x g at 4 °C for 35 minutes.

The cleared cell lysate was loaded onto two 5 mL HiTrapQ HP anion exchange columns connected in tandem using an Akta FPLC (GE Healthcare). The protein was purified by anion exchange chromatography as described previously[19,55]. Following the anion exchange column, the protein was loaded onto a Superdex200 size exclusion column. The protein that eluted from the column was collected, and was concentrated in an Amicon filter centrifuge tube (10,000 molecular weight cutoff) to a final concentration of about 10 µM. This protein was flash frozen in liquid nitrogen and stored under liquid nitrogen.

The concentration of protein was estimated based on the absorption of the sample at 452 nm, using an extinction coefficient of 11,300 M^{-1} cm^{-1} [55]. The total protein yield was about 0.2 mg of protein per gram of cell paste. The purity of the isolated protein was analyzed on a denaturing polyacrylamide gel. The concentration of protein with bound flavin mononucleotide cofactor was estimated from the ratio of the absorption of the sample at 274 nm to the absorption of the sample at 452 nm. The ratio of Abs_{274}/Abs_{452} was typically ~7, which was higher than the ratio of 4.6-4.8 reported for the oxidized form of this protein[55], suggesting the presence of some apo or partial reduced forms of the flavodoxin.

Preparation of Flavodoxin II for Electrochemistry Experiments. Prior to electrochemistry experiments, sodium dithionite was removed from all protein samples using a PD10 column (GE Healthcare). This procedure was performed in a McCoy anaerobic chamber with a 95 % / 5 % Ar / H_2 atmosphere. The column was equilibrated in five column volumes of electrochemistry buffer (50 mM potassium phosphate, pH 7.5, 150 mM NaCl) with 10 mM sodium dithionite. This step allowed for the reduction of reactive oxygen species prior to introduction of the protein to the column. Then, the column was equilibrated in five column volumes of electrochemistry buffer to remove all sodium dithionite. All buffers were filtered with a 0.2 micron filter, and oxygen was removed from the buffer by iterative cycles of vacuum followed by filling with argon. The protein was exchanged into electrochemistry buffer using the PD10 column. UV-visible absorption spectroscopy was used to quantify protein concentration, and to determine the extent of protein oxidation during the course of sample preparation. Following this procedure, the protein was flash frozen in liquid nitrogen, and when necessary was transported under liquid nitrogen.

Flavodoxin II Kinetics on DDAB-Modified BPGE. The kinetics of the redox process observed on DDAB-modified BPGE were studied to determine if flavodoxin II diffused in the DDAB films on the time scale of voltammogram acquisition. Only the robust, lower potential species was monitored with this method. The signal intensity of the lower potential redox process observed in these films was proportional to the square root of the scan rate **(Figure S3C)**. Thus, the redox process occurring at the electrode was diffusion-limited, suggesting that flavodoxin II was diffusing in the surfactant film. This result was consistent with the kinetics observed for other proteins, like myoglobin and cytochrome c [30]. However, there are also examples of proteins that are immobile in surfactant films under the conditions assayed in the described experiments, like hemoglobin and cytochrome P450 [33]. The Randles-Sevcik equation could be used to estimate the diffusion constant of flavodoxin II in surfactant films as $6.4 \times 10^{-8} \pm 5.1 \times 10^{-8}$ cm^2/s. This estimate assumes that the protein concentration is about the same in solution as in the surfactant film, and that it is a one electron redox process. This diffusion rate is about ten times smaller than the diffusion coefficient of $7.4 \pm 0.9 \times 10^{-7}$ cm^2/s for the *A. vinelandii* flavodoxin reported for glassy carbon electrodes in the presence of the promotor neomycin [21], which in turn is close to the value 1.3×10^{-6} cm^2/s calculated for the diffusion constant for a sphere the size of flavodoxin II undergoing Brownian motion in solution. These observations suggest that interaction with the DDAB surfactant impedes the free diffusion of flavodoxin II at the electrode surface.

Figure S1. Cyclic Voltammogram of Flavodoxin II on Unmodified Basal Plane Graphite Electrodes. The basal plane graphite electrode (BPGE) was placed in a 200 μM solution of flavodoxin II in 50 mM potassium phosphate, pH 7.5, 150 mM NaCl, and a cyclic voltammogram was acquired at a scan rate of 50 mV/s (gray). A basal plane graphite electrode was modified with 10 mM DDAB. This electrode was placed in a 200 μM solution of flavodoxin II in 50 mM potassium phosphate, pH 7.5, 150 mM NaCl, and a cyclic voltammogram was acquired at a scan rate of 50 mV/s (black).

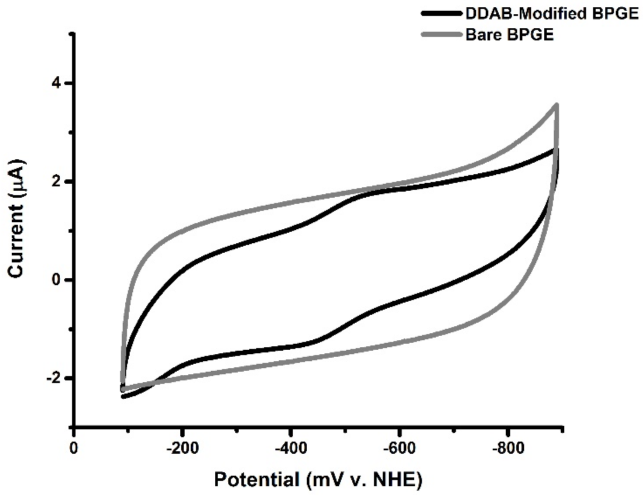

Figure S2. Cyclic Voltammogram of FMN. The basal plane graphite electrode was placed in a 200 μM solution of FMN in 50 mM potassium phosphate, pH 7.5, 150 mM NaCl, and a cyclic voltammogram was acquired at a scan rate of 20 mV/s. The higher potential peak on the voltammogram had a midpoint potential of -138 ± 3. The lower potential peak on the voltammogram had a midpoint potential of -303 ± 1. The scan rate dependence of the signal intensity for free FMN in solution indicated that this molecule diffused in the DDAB films at a diffusion rate of $5.7 \times 10^{-7} \pm 1.4 \times 10^{-7}$ cm^2/s. This calculation assumed that the concentration of FMN was the same in solution as it was in the surfactant film, and that the redox process observed was a two electron process.

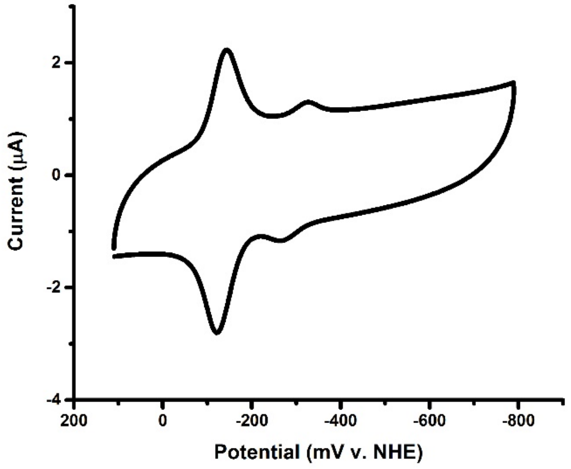

Figure S3. Cyclic Voltammogram of Flavodoxin II with the Higher and Lower Potential Species. (S3A) The electrode was soaked in 200 µM authentic *A. vinelandii* flavodoxin II in 50 mM potassium phosphate, pH 7.5, 150 mM NaCl for 20 minutes. The electrode was transferred to 10 mM phosphate buffer, pH 7, and a cyclic voltammogram was acquired at a scan rate of 20 mV/s. Using this method, the midpoint potential of E_1 was measured as -454 ± 7 mV, and the midpoint potential of E_2 was measured as -164 ± 12 mV. **(S3B)** Square wave voltammogram of flavodoxin II on DDAB-modified basal plane graphite electrodes showing the reduction potential of E_1 and E_2. **(S3C)** Dependence of the cathodic peak signal intensity on the scan rate for the lower potential peak of the cyclic voltammogram of flavodoxin II on DDAB-modified basal plane graphite electrodes. The linear dependence of the current on the square root of the scan rate indicates that the redox process at the electrode is diffusion limited.

S3A

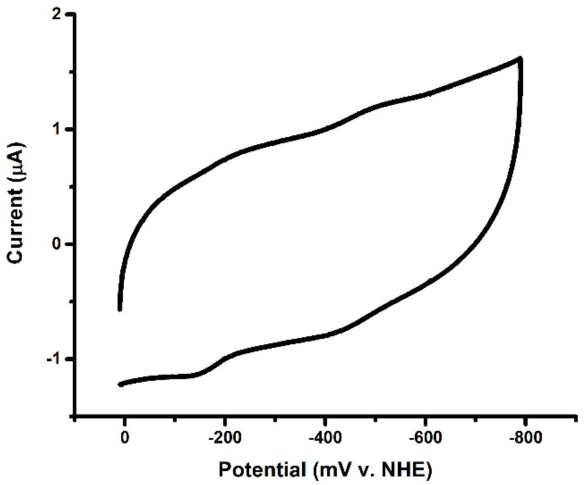

S3B

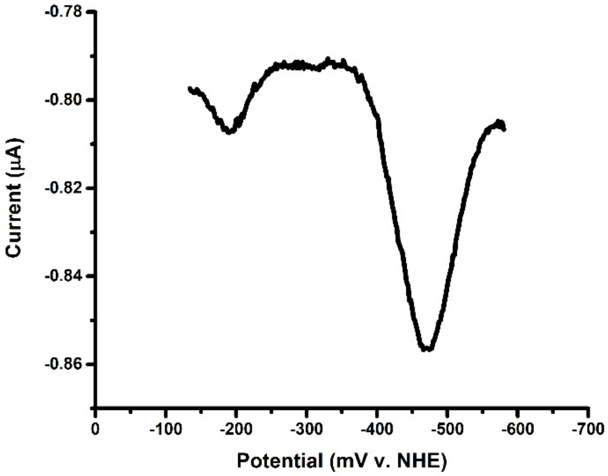

S3C

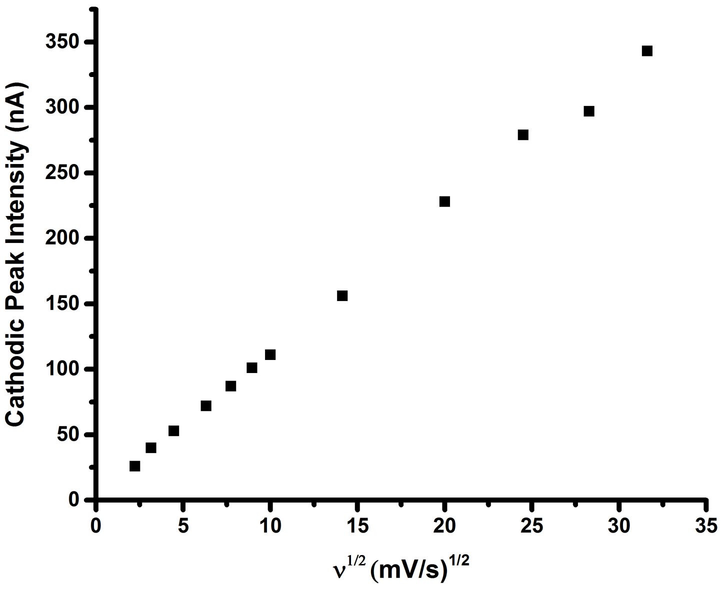
Cathodic Peak Intensity (nA)
0
50
100
150
200
250
300
350
0
5
10
15
20
25
30
35
ν1/2 (mV/s)1/2

Figure S4. Model of the Fe-Protein MoFe-protein Complex generated with ClusPro 2.0. The Fe-protein structure used to generate the model was PDB 4WZB. The MoFe-protein structure used to generate the model was PDB 3U7Q. The Fe-protein is shown in white with the 4Fe-4S cluster highlighted in purple. The MoFe-protein is shown in teal. The P-cluster and the FeMo-cofactor are highlighted in orange.

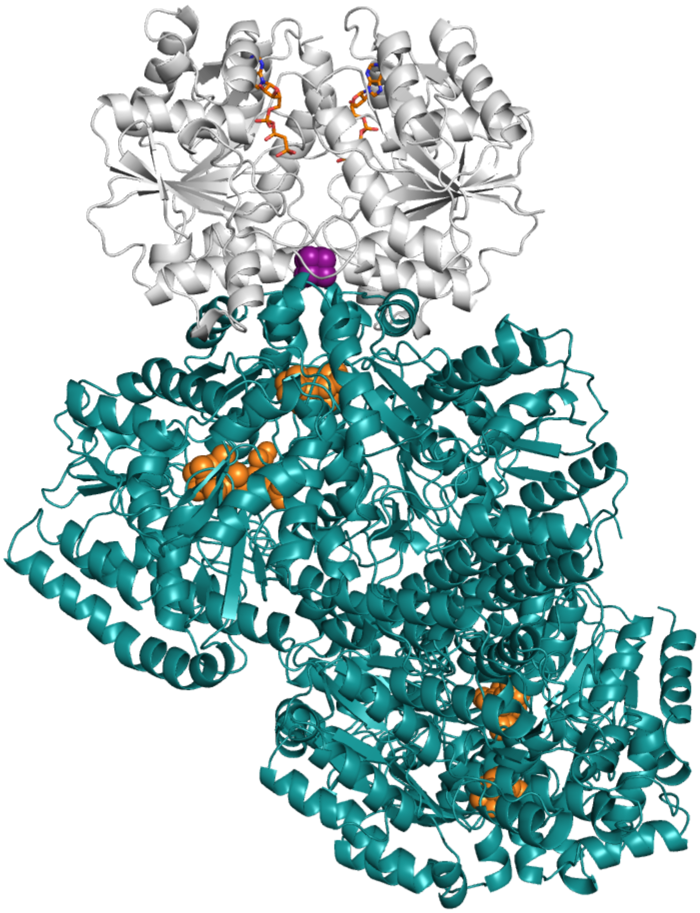

Table S1. Midpoint Potentials of *Azotobacter vinelandii* Flavodoxin II from the Literature

E_2 (mV v. NHE) *(pH)*	E_1 (mV v. NHE) *(pH)*	Electrochemical Method	Reference
-224 *(pH 8)*	-458 *(pH 8)*	Redox titration with chemical mediators (UV-visible absorption spectroscopy)	(22)
-245 *(pH 8)*	-515 *(pH 8)*	Microcoulometry at Controlled Potentials (UV-visible absorption spectroscopy)	(27)
-270 *(pH 7.7)*	-464 *(pH 7.7)*	Redox titration with chemical mediators (UV-visible absorption spectroscopy)	(28)
+50 *(pH 8.2)*	-495 *(pH 8.2)*	Redox titration with chemical mediators (UV-visible absorption spectroscopy)	(29)
--	-500 ± 10 *(pH 7)**	Redox titration with chemical mediators (UV-visible absorption spectroscopy)	(12)
--	-449 *(pH 6)*<br>-488 *(pH 8.5)*	Direct electrochemistry on glassy carbon modified with neomycin (Cyclic voltammetry)	(21)
-74 ± 10 *(pH 6)*<br>-183 ± 10 *(pH 8.5)*	-446 ± 10 *(pH 6)*<br>-468 ± 10 *(pH 8.5)*	Redox titration with chemical mediators (EPR spectroscopy)	(21)

*Measurement not accurate due to mediator system chosen

Table S2. Data Collection and Refinement Statistics for *A. vinelandii* Flavodoxin II (*nifF*). Values in brackets represent the highest resolution shell

**Data collection statistics**	**Flavodoxin II**
Wavelength (Å)	0.99987
Resolution range (Å)	35.73 – 1.17 (1.24 – 1.17)
Unique reflections	56,103 (7,613)
Completeness (%)	99.2 (95.0)
Multiplicity	18.4 (15.3)
Space group	$P3_121$
Unit cell parameters	
a, b, c	39.87, 39.87, 178.66
α, β, γ	90, 90, 120
R_{merge}	0.069 (0.686)
$R_{p.i.m.}$	0.016 (0.177)
$CC_{1/2}$	0.999 (0.885)
Mn (I / σ(I))	24.8 (4.4)
**Refinement statistics**	
R_{cryst} (%)	14.95
R_{free} (%)	16.76
r.m.s.d. bond lengths (Å)	0.010
r.m.s.d. bond angles (°)	1.130
Average B-factor ($Å^2$)	17.50
Ramachandran: allowed (outliers) (%)	100 (0)
